# Multiple Causes for Delay in Arrival at Hospital in Acute Stroke Patients in Aydin, Turkey

**DOI:** 10.1186/1471-2377-8-15

**Published:** 2008-05-13

**Authors:** Sakine Memis, Emel Tugrul, E Didem Evci, Filiz Ergin

**Affiliations:** 1 Asist. Prof. Dr., Department of Medical Nursing, Adnan Menderes University, School of Health Aydin, Turkey; 2 Department of Fundamentals of Nursing, Adnan Menderes University, School of Health Aydin, Turkey; 3 Asist. Prof. Dr., Department of Public Health, Adnan Menderes University, School of Medicine Aydin, Turkey

## Abstract

This descriptive, hospital-based study, performed in western Turkey, was designed to assess the level of pre-hospital delay and reasons for such delay in acute stroke patients, taking into consideration certain factors such as socioeconomic status, availability of transport options at onset of symptoms. Data were collected from hospital records, and a questionnaire was administered that included questions about socio-demographics, self-reported risk factors and questions related to hospital arrival. The rate of patients arriving at the hospital more than 3 hours after symptom onset was found to be 31.6% for this study. Approximately 1/3 of patients delayed going to the hospital because they were waiting for symptoms to go away while 1/3 of patients were not aware of the importance of seeking immediate medical help. There was a significant relationship between the use of ambulance transportation and length of time before arrival at the hospitals, though there was no statistically significantly relationship between the existence of stroke risk factors and hospital arrival delay. These results will likely be helpful to health care decision makers as they develop a model for stroke health care and community based training.

## Background

Stroke is one of the leading causes of mortality of adults in Turkey as it is in many countries [1]. The importance of early intervention (such as recombinant tissue plasminogen activator (rt-PA) within 3 hours of onset of stroke) in acute stroke situations is well known to prevent permanent disability and mortality otherwise caused by stroke [2-5]. However, several studies report that certain factors such as socioeconomic status (low socioeconomic status increase or no difference on effect on delay in acute ischemic stroke), availability of transport options (the use of helicopters decreases transport times and helps rapid access to the treatment facilities), living alone or being alone at onset of symptoms (increase effect on delay in acute ischemic stroke) play a role in late arrival at hospitals thereby decreasing the ultimate success of medical interventions for stroke patients [6-8]. There is little research about awareness and knowledge level concerning stroke and also about the structure and availability of stroke-related health services (stroke unit and related support systems) in Turkey. There is no national stroke-monitoring service or database. This study is designed to assess the level of pre-hospital delay and reasons for such delay in acute stroke patients. Results from this study will likely be helpful to Turkish health care decision makers as they work toward the development of a model for stroke health care and community based training about stroke awareness and the importance of early intervention.

## Methods

This is a descriptive hospital-based study conducted between January and November 2005, in Aydin (population, 217,558), a city in Western Turkey. There are two main governmental hospitals in the city center of Aydin. These hospitals accept all patients from Aydin and neighboring cities. A distance from urban and rural areas to the hospitals varies from 10 km to 115 km. Aydin is an affluent city and is located on a grassy plain where there are no traffic jams. The study was performed at two hospitals which are centrally located and see a significant number of stroke patients. In 2005, a total of 302 stroke patients (57 at the University hospital, 245 at the State hospital) were admitted in these two hospitals. Ninety-eight (32%) of these patients participated to this study. The remaining 68% were not eligible to participate. This study was approved by The Hospital Directorate which is legally responsible for hospital management. The researchers followed the ethical principles of the Declaration of Helsinki.

Verbal informed consent was obtained from all subjects. In Aydin, there are not any spesific stroke units in the hospitals. For this reason, patients in the study were identified from daily records of admitted patients in these hospitals. Data were collected from the emergency departments, neurology services and neurology intensive care units by the researchers. A questionnaire was administered to patients, their relatives and doctors utilizing face-to-face interviews and data were also collected from medical records. Two specialist nurses who were certificated by the Turkish Society of Cerebrovascular Diseases on stroke collected data by taking notes during the interviews and by reviewing the medical records of patients. Inter-reliability of the researchers was established during four hours of training on the study and questionnaire and pre-testing of the questionnaire. Three exclusion criteria were determined as "unconsciousness, aphasic (total, Wernike, Broca) and refusal of patient and/or family". However, as a limitation of the study, percentage of patients excluded according to above mentioned criteria were not recorded by the researchers.

The questionnaire was developed in accordance with relevant literature. Questions which are commonly used in hospitalized stroke patients interviews were chosen for use in the questionnaire to maintain validity. It was pre-tested on ten patients who were not included in the study, and was modified based on the pilot results [9-14]. The questionnaire contained 21 questions in four sections. The first section included seven close-ended questions about socio-demographics including age, sex, marital status, education, occupation, social insurance (which includes health care coverage) and place of residence. The second section included seven "yes/no" questions evaluating self-reported risk factors (high blood pressure, heart disease, previous stroke, diabetes, high cholesterol, smoking, alcohol). When patients reported that they had at least one stroke risk factor in response to a question delineating these, one point was given. If none were present, the score was recorded as "zero". The third section of the questionnaire included five questions related to hospital arrival. Two were open-ended questions: i) "Please determine the time of onset of stroke"; ii) "What was the time interval from stroke onset to hospital arrival?" Three questions (close-ended) were asked about arrival time (day of the week and time of the day) and type of transportation used. The last section included two open-ended questions: i) "Why did you choose to go to the hospital?"; ii) "What were the main reasons for any delay in your arrival at the hospital?". SPSS 11.0 for Windows^® ^software was used for statistical analysis of the data. The descriptive data were presented as median, mean ± standard deviation (SD) and percentages. Patients were categorized into two groups depending on length of time of symptom onset to arrival at the hospital (≤ 3 hours and > 3 hours). The time of symptom onset was defined as the moment when symptoms started or the moment the patients or relatives first noticed warning signs. If the stroke began when the patient was asleep, the time when they awakened was considered the time of symptom onset. Length of time of symptom onset to arrival at the hospital was classified into two groups as ≤ 3 hours and > 3 hours according to the results of previous studies [4,9,10,13,15,16]. Hospital arrival delay was determined based on patients' or relatives' interview or extracted from the medical chart.

These two groups (≤ 3 hours and > 3 hours) were then compared regarding their responses to the four sections of questions. Chi-square test, Fisher's exact test and t-test were used for analytical evaluation. The differences were considered to be statistically significant when p < 0.05. The open ended comments were summarized as percentages.

## Results

This study was carried out with 98 patients. Fifty three patients were female and the mean age of patients was 67.7 ± 11.0 (40–92). Detailed socio-demografic features of the patients are shown in Table 1. When patient were read a list of stroke risk factors 91 patients answered affirmatively that they had at least one stroke risk factor. Reported risk factors were as follows: high blood pressure (69.4%), heart disease (43.8%), diabetes (38.8%), smoking (30.6%), previous stroke (27.6%), high cholesterol (21.4%), alcohol (5.1%) and obesity (4.1%). It was determined that 67 patients (68.4%) arrived at the hospitals within three hours and 31 patients (31.6%) arrived at the hospitals after 3 hours from the onset of stoke symptoms.

**Table 1:** Socio-demographics Features of Patients, Aydin, 2005

	***n***	***%***
**Age (year), mean ± SD**	(n = 98) 67.74 ± 11.03	
**Sex**		
Female	53	54.1
Male	45	45.9
**Residence**		
Urban	24	24.5
Rural	74	75.5
**Education**		
Illiterate	58	59.2
Literate	40	40.8
**Marrital status**		
Married	64	65.3
Single+widow	34	34.7
**Occupation**		
Self-employed	33	33.7
Paid work	12	12.2
Unemployed	53	54.1
**Social Security (Public Health Insurance)**		
Yes	88	89.8
No	10	10.2

Eighty one of the patients (82.6%) reported to the hospitals on a weekday and 73 (74.4%) arrived during daytime hours. Sixteen of the patients (16.3%) arrived at the hospital by ambulance. The most common stroke warning signs experienced by 79.6% of patients were sudden trouble walking, dizziness, loss of balance or coordination. The second most common sign, reported by 75.5% of patients, was sudden weakness or numbness of the face, arm or leg, especially on one side of the body. Other warning signs were sudden confusion, trouble speaking or understanding (74.5%), sudden vision changes (10.2%) and sudden severe headache with no known cause (10.2%). These stroke warning signs were classified according to information provided from "American Stroke Association" documents. The most common reason patients stated for delay was waiting for symptoms to go away (35.5%). Other reasons for delay and associated percentages are given in Table 2. There was a significant relationship between length of time prior to arrival at the hospitals (within three hours) and type of transportation used or available.

**Table 2:** Reasons Reported by Patients for pre-hospital delay, Aydin, 2005

**Reasons**	**n**	%*
Waiting for symptoms to go away	11	35.5
Not realizing the urgency of seeking medical help	10	32.3
Stroke while sleeping	6	19.4
Not able to call for help	4	13.3
Taking medicine and waiting for it to take effec	2	6.5
Calling doctor to come to the home	1	3.2

*%s are taken from stated reasons because patients were allowed to cite more than one reason for pre-hospital delay.

Patients who arrived within 3 hours of onset of symptoms were more likely to come by ambulance (22.4%) or private vehicle (77.6%) (χ^2 ^= 5.697, p < 0.05). Age, gender, education, marital status, occupation, social insurance (which includes health care coverage), place of residence, self-reported risk factors of stroke, day of the week or time of day had no effect on length of time before arrival at the hospitals (p > 0.05) (Table 3). There was no statistically significant relationship between experiencing stroke warning signs and length of time before arrival at the hospitals (p > 0.05).

**Table 3:** Factors Influencing Early Admission of Patient to the Hospital (≤ 3 hours and > 3 hours), Aydin, 2005

**Factors**	≤ **3 hours**		**> 3 hours**			
	***n***	***%***	***n***	***%***	**χ^2^**	**P**
**Age (year), mean ± SD**	(n = 67)	66.50 ± 11.56	(n = 31)	70.38 ± 9.40	-1.633*	0.106
**Gender**						
Female	38	71.7	15	28.3	0.592	0.442
Male	29	64.4	16	35.6		
**Residence**						
Urban	16	66.7	8	33.3	0.043	0.837
Rural	51	68.9	23	31.1		
**Education**						
Illiterate	38	65.5	20	34.5	0.534	0.465
Literate	29	72.5	11	27.5		
**Marrital status**						
Married	43	67.2	21	32.8	0.119	0.730
Single+widow	24	70.6	10	29.4		
**Occupation**						
Self-employed	21	63.6	12	36.4	0.629	0.730
Paid work	8	66.7	4	33.3		
Unemployed	38	71.7	15	28.3		
**Social Security (public health insurance)(social insurance)**						
Yes	61	69.3	27	30.7	0.361	0.721
No	6	60.0	4	40.0		
**Self-reported stroke risk factors**						
Yes	62	68.1	29	31.9	0.033	1.000
No	5	71.4	2	28.6		
**Type of transport to the hospital**						
Personal car/public trans.	52	63.4	30	36.6	5.697	0.017
Ambulance	15	93.8	1	6.3		
**Day of the week**						
Weekday	55	67.9	26	32.1	0.047	0.829
Weekend	12	70.6	5	29.4		
**Time of the day**						
Night	18	72.2	7	28.0	0.205	0.651
Daytime	49	67.1	24	32.9		

* %s presented as line percentage ** t value is given

## Discussion

Although the rate of patients arriving at the hospital's stroke unit more than 3 hours after symptom onset has previously been reported as high as 50–70% in other series and in many Western countries at least 50% patients arrive within 6 hours, this study reports this rate to be at 31.6% [14-17]. This is likely due to the fact that Aydýn is a small, affluent city in Western Turkey with no traffic jams and that the longest distance to the hospital from rural areas is 115 km. In addition to this reasons, it might be related with differences in the definition of symptom onset in the literatures. Most studies of stroke delay define symptom onset as the moment when symptoms started or the moment the patients or relatives first noticed warning signs [9,11,14-17]. Only one study was achieved to define as the time the subject was last known symptom free [13]. Since perhaps 1/3 of patients have their stroke while they are asleep, the definition used could easily affect the percentage of patients in < 3 hour group. On the other hand, there may have been substantially more patients who would have been classified as delaying, if 2 hours or less had been chosen. Approximately one third of patients delayed going to the hospital because they were waiting for symptoms to go away. Additionally, 1/3 of patients were not aware of the urgency for seeking immediate medical help and therefore delayed seeking such help in the first three hours, which is imperative for rt-PA administration. In early 2006 rt-PA administration was initiated for stroke treatment in the Turkish medical system. Lack of awareness of the urgency of stroke symptoms was shown to be associated with failure and/or difference in the perception of the importance or severity of symptoms in other studies [16,18]. Waiting for symptoms to go away has previously been defined as "natural coping response to indecision" [6]. A statement from the American Heart Association Council on Cardiovascular Nursing and the Stroke Council states that there was no ongoing study concerning the above mentioned topic [6]. Other studies have found that the belief that symptoms were not of a serious nature, incorrect perception of the severity of the symptoms and decision to wait for symptoms to go away affected the length of time before patients went to the hospital [13,19]. In this study, a significant relationship was found between the use of ambulance transportation and length of time before arrival at the hospitals. However, only 16 of 98 patients (16.3%) arrived by ambulance. This is a low percentage of use of the emergency system for transport in Aydin even though there is a well organized emergency service network called 112 (access to Emergency Services, similar to the US 911 system). Some published studies in other countries indicate that the percentage of patients who arrive at the hospital by ambulance is 35–46% among stroke patients [11,15]. In the questionnaire in this study, there was no question asked about reasons for this low percentage. It would be useful to design detailed studies to explain the low percentage of usage of 112 in Aydin in the future.

It is difficult to explain the high rate of self-reported stroke risk factors in this study in comparison with other studies in the literature. Objective data from the medical records (prior medical treatment, calculation of BMI, etc.) would have been a more reliable data source but the records were incomplete. In this Aydin study as in other published studies, high blood pressure is the most prevalent risk factor for stroke, with heart disorders being second [19,20]. There was no statistically significantly relationship between stroke risk factors and length of time before arrival at the hospital. These results are notable since just seven patients reported no risk factors. It is expected that patients, who are knowledgeable about stroke risk factors and who report at least one risk factor would seek expert stroke care in a relatively short period of time. However, Derex et al. suggested that there is no relationship between awareness of risk factor and length of time before arrival at the hospital [15]. In addition the patients in this study may not have been aware that their medical condition (hypertension, for example) was a risk factor for stroke. In thisstudy, most of the participants (67%) reached the hospital within 3 hours of symptom onset, however one third of the patients arrived three hours after symptom onset, indicating there is room for improvement. The rate of ambulance usage is very low among this patient population. We believe that it is important to increase awareness of stroke symptoms and to further educate the public about the appropriate use of 112 (access to Emergency Services, similar to the U.S. 911 system). Awareness of stroke symptoms and risk factors must be in the forefront of health promotion programs such as the Massachusetts Department of Public Health's "ACT FAST" campaign and some initiatives of the World Health Organization (WHO) such as promoting physical activity and reversing the obesity epidemic [21,22].

## Conclusion

This is the first descriptive hospital-based study on strokes performed in Turkey. Though this is a local study and the sample size is small, the collected data can be helpful in the creation of a stroke database for Turkey and can be utilized in the international literature as a country sample. There are methodological difficulties that need to be discussed and clarified. One of the main issues is the small sample size (32%; 98 of 302 patients). These 98 stroke patients may not be representative of the whole population and should be considered when comparing these results with other studies. For a study of this kind, better information and record systems need to be established, kept under control and evaluated frequently.

In this study 68.4% of patients arrived at the hospitals within three hours of symptoms onset. We had to identify patients from daily records of admitted patients in these hospitals and collect data from departments by ourselves. We preferred to use interview questions used for general examinations in the hospital and also parallel to the references to catch common language during interviews with health staff and patients in the hospital, during working time and sensitive period of a serious health problems.

In this study, approximately one third of patients were waiting for symptoms to go away and also one third of patients were not aware of the urgency for seeking immediate medical help and therefore they lost the first three hours, which is imperative for rt-PA administration. To reduce this rate, health promotion programs need to focus on awareness of stroke symptoms and risk factors for not only medical experts but also for the public

## Competing interests

The authors declare that they have no competing interests.

## Authors' contributions

SM and EDE made substantial contributions to the conception and design, acquisition of data, and analysis and interpretation of data; FE was the adviser for statistical evaluation; and all authors were involved in drafting the manuscript or revising it critically for important intellectual content; and SM and EDE have given final approval of the version to be published. All authors read and approved the final manuscript. Each author has participated sufficiently in the work to take public responsibility for appropriate portions of the content.

## Pre-publication history

The pre-publication history for this paper can be accessed here:


